# Influence of substrate on the structure of predominantly anatase TiO_2_ films grown by reactive sputtering

**DOI:** 10.1039/c7ra10974a

**Published:** 2018-02-14

**Authors:** Iuri S. Brandt, Cristiani C. Plá Cid, Carlos G. G. Azevedo, André L. J. Pereira, Luana C. Benetti, Andre S. Ferlauto, José H. Dias da Silva, André A. Pasa

**Affiliations:** Laboratório de Filmes Finos e Superfícies, Universidade Federal de Santa Catarina Florianópolis 88040-900 Brazil iuri.brandt@pgfsc.ufsc.br andre.pasa@ufsc.br; Programa de Pós-Graduação em Ciência e Engenharia de Materiais, Universidade Federal de Santa Catarina Florianópolis 88040-900 Brazil; Faculdade de Ciências, Universidade Estadual Paulista UNESP Bauru SP 17033-360 Brazil jhdsilva@fc.unesp.br; Universidade Federal da Grande Dourados UFGD Dourados MS 79804-970 Brazil; Departamento de Física, Universidade Federal de Minas Gerais Belo Horizonte 31270-901 Brazil

## Abstract

TiO_2_ films are grown on LaAlO_3_ (001), Si (100) and SiO_2_ substrates by reactive radio frequency sputtering. X-ray diffraction (XRD) pole figures revealed important characteristics about the texture and phase distribution on those films. Combined with spectroscopic ellipsometry, the pole figures allowed the analysis of the growth characteristics over the whole volume of the layers. Details in the microstructure of the films were probed using transmission electron spectroscopy. Anatase is the dominating phase in the films grown on all substrates. On TiO_2_/LaAlO_3_ fims, the mosaicity is very low, so that the pole figure closely resembles that of anatase monocrystals. Detailed inspection of XRD pole figures reveals a small amount of rutile in the TiO_2_/LaAlO_3_ films. For the growth of TiO_2_ onto SiO_2_, rutile and brookite phases are also detected. Transmission electron microscopy and XRD results show the formation of anatase {112} twins in films grown on the different substrates, suggesting that the anatase {112} twin mediates the growth of rutile and brookite phases. Optical results are in agreement with the XRD observations: the optical properties of TiO_2_/LaAlO_3_ films are similar to the ordinary values of bulk anatase crystals, indicating the orientation of the film in the [001] direction, whereas results for TiO_2_/SiO_2_ are compatible with lower crystalline ordering.

## Abstract 2

Revealing the crystalline structure of predominantly grown anatase TiO_2_ films.

## Graphical abstract

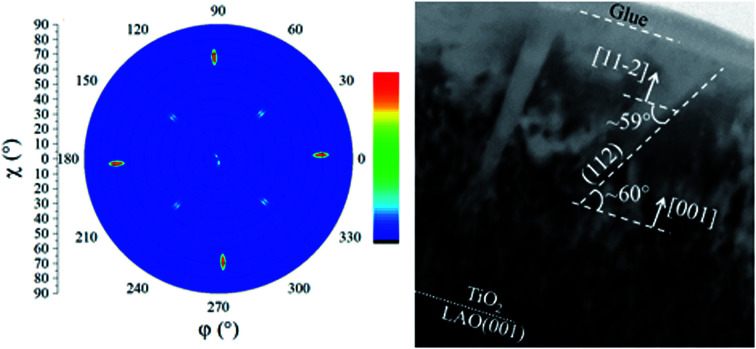

## Introduction

Titanium dioxide (TiO_2_) is a wide band gap semiconductor that has been intensively studied since the observation of water photolysis on its surface in 1972.^1^ Nowadays, TiO_2_ is the most widely used oxide for photocatalysis of organic pollutants.^2–4^ In past years, new applications in the fields of biocompatibility,^5^ self-cleaning processes,^6^ photovoltaics,^7^ lithium ion batteries^8^ and high-*k* gate dielectrics^9^ have reinforced the needs for research on TiO_2_ film deposition methods. The performance of these films in such technologies strongly depends on the TiO_2_ polymorphic phase employed: rutile, anatase or brookite. Anatase exhibits advantages over rutile for catalysis, photocatalysis and solar cell applications.^2,10–12^ However, in some cases, due to synergistic effects, a phase blend is expected to enhance the photocatalytic activity.^13^ Moreover, the ability to control the texture and the crystallinity of the TiO_2_ films allows increases in efficiency of lithium batteries or of catalysis processes.^14–19^

Depending on deposition method and substrate nature, pure^20–23^ or mixed-phase^24,25^ TiO_2_ films can be obtained. Although anatase^20–22,26–28^ and brookite^29^ are metastable phases, they can be grown in pure form on single crystal substrates *via* epitaxial stabilization. It is important to determine the relative concentration of each phase, the presence of defects and the predominant crystalline orientation in TiO_2_ films, in order to better understand and control their growth as well as to better understand the correlation between growth processes and the optical and electronic properties of the corresponding films.

The best epitaxy of the anatase TiO_2_ occurs when lanthanum aluminate LaAlO_3_ (001), labeled simply as LAO, is used as substrate.^26,28,30–32^ This is due to the very low lattice mismatch (0.2%).^26^ The pulsed laser deposition (PLD) is by far the most used technique for this kind of growth,^28,32^ followed by the molecular beam epitaxy (MBE) growth.^26,27,33^ While these techniques offer excellent control of the growth conditions, they have the limitations of depositing in small areas, and high costs. In an attempt to develop alternatives, e-beam evaporation was tested^31^ and a few groups have considered the use of reactive sputtering deposition.^20,30,34^ These techniques are simpler, and present lower cost than PLD and MBE. Besides, the reactive sputtering has the potential to produce uniform growth over very large areas.^35^ This makes the technique attractive for several technological applications, but it still needs further understanding and development.

In this report, we investigate the growth of TiO_2_ films by reactive sputtering and the dependence of phase formation and film crystallinity on the nature of the substrate. The TiO_2_ layers were grown onto the (001) surface of LAO, Si (100) and fused silica substrates. Local details in the structure of the films, including twin formation, are analyzed using transmission electron spectroscopy. X-ray diffraction (XRD) pole figures were used for the first time to analyze this epitaxy. They revealed important characteristics about the texture and phase distribution on those films. The joint use of spectroscopic ellipsometry and pole figures allowed the probe of the growth characteristics over the whole volume of the samples.

## Experimental

The films were deposited by radio frequency (RF) magnetron sputtering using a 75 mm diameter metallic Ti target (99.999%) in an Ar + O_2_ atmosphere (99.9999%). The deposition RF power was 120 W, using a KJL Torus magnetron cathode. The total pressure was 5.0 × 10^−3^ Torr, using Ar flow of 40.0 sccm, while the oxygen flow was 0.2 sccm. The residual pressure was below 1.0 × 10^−6^ Torr. Fused silica (a-SiO_2_), Si (100) and LAO (001) substrates, kept at 450 °C, were used simultaneously in the depositions. As no treatment to remove the native oxide on the top of the Si (100) was performed, in what follows we refer to this as the SiO_2_/Si(100) substrate. It was observed that XRD results were identical for samples deposited onto SiO_2_/Si(100) and a-SiO_2_ substrates, so we have shown here just the ones corresponding to TiO_2_ films deposited onto SiO_2_/Si(100).

Samples deposited under continuous and interrupted O_2_ flow (20 interruptions per deposition) were analyzed. Further details about sample preparation can be found elsewhere.^24^

Structural properties were investigated by XRD with CuK_α1_ radiation (1.54 Å). The pole figures for in-plane and out-plane orientations were performed by electing a diffraction angle, 2*θ*, with the tilt angle, *χ*, varying from 0 to 90° and the azimuthal angle, *ϕ*, varying from 0 to 360°, for each value of *χ*. In order to obtain structural information associated to a few grains, a 200 kV-TEM (JEOL JEM-2100) was used in bright and dark field imaging modes. Additionally, selected electron area diffraction (SAED) technique was also employed.

Optical characterizations of the films were performed using transmittance and reflectance measurements and spectroscopic ellipsometry. The transmittance measurements at normal incidence and reflectance at nearly normal (8°) were performed in a Perkin Elmer Lambda 1050 spectrophotomer, with 150 mm integrating sphere apparatus, in the 200–2500 nm range. Spectroscopic ellipsometry measurements were performed using a M-2000 J. A. Woollam spectrometer in the 246–1690 nm range.

## Results and discussion

### LAO substrate

**Fig. 1:**
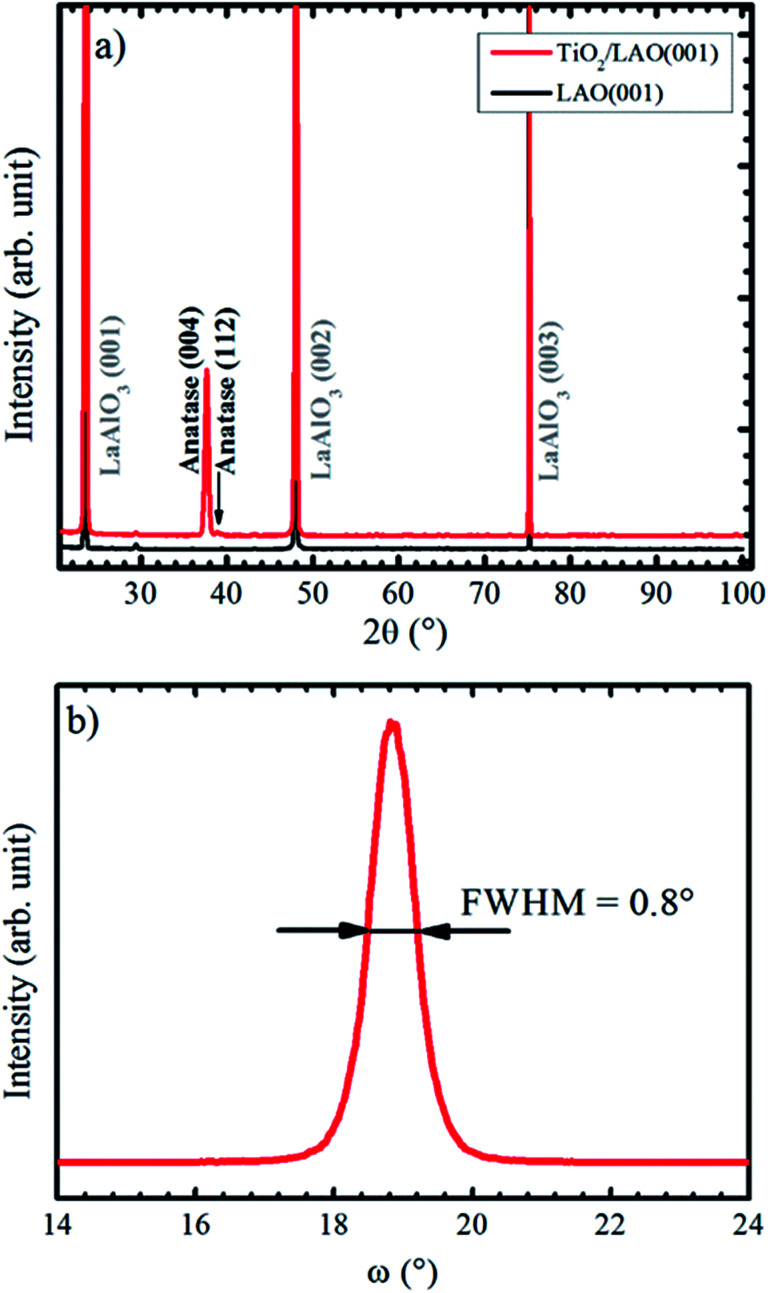
(a) XRD Bragg–Brentano pattern recorded for LAO and TiO_2_ film grown onto LAO. The arrow indicates the anatase (112) peak position. In (b) it is shown anatase (004) XRD rocking curve.

X-ray diffractograms for the TiO_2_/LAO sample deposited using continuous oxygen flow are shown in Fig. 1. Results on twin samples prepared under modulated oxygen flow display identical results.

The large domination of the (004) peak in the Bragg–Brentano diffractogram of Fig. 1a indicates that the TiO_2_ anatase phase preferentially grows with the [001] direction perpendicular to the LAO substrate surface. Such preferential growth in anatase [001] direction is in agreement with previous reports.^36,37^ A low intensity peak, close to the anatase (004) and indicated by an arrow in Fig. 1a, is also observed and assigned to anatase [112] direction. All other XRD peaks come from the substrate. Fig. 1b displays a rocking curve of the anatase (004) peak, with full width at half maximum (FWHM) equal to 0.8°. This value is comparable to the ones for thick TiO_2_ films obtained by pulsed laser deposition,^38^ which is a well-recognized technique for growing high quality films (see for instance the book by D. B. Chrisey and G. K. Hubler^39^ and references therein).

Lotnyk and co-workers^31^ analyze the growth of anatase films onto SrTiO_3_ and LAO using reactive e-beam evaporation. Substrate temperatures in the 500−1000 °C range were used during the growth (while the substrates were kept at 450 °C in our results). They found that the film–substrate interface is composed by anatase (001) and LAO (001) surfaces, similarly to the present conditions. The full width at half maximum of the rocking curve peaks attained by their growth at 1000 °C on LAO (0.2°) is smaller than the one (0.8°) reported on Fig. 1b. This difference is probably due to the higher substrate temperatures, which promotes ordering of crystallites, as opposed to the bombardment of the substrate by energetic particles during sputtering growth. The incidence of energetic particles from the plasma on the growth surface is likely to produce defects and higher strain effects on the crystallites. The comparison suggests that the sputtering growth at lower power and higher substrate temperatures than the used in the present report should produce higher ordering in the films.

**Fig. 2:**
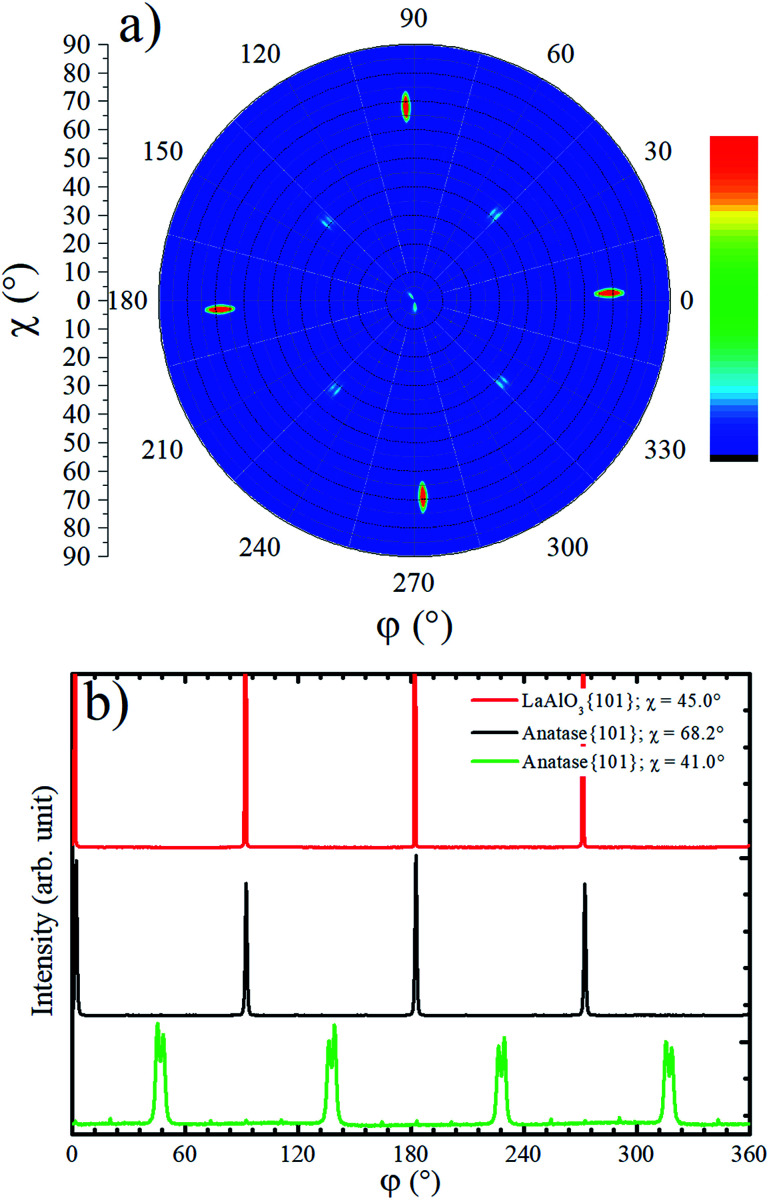
(a) Anatase {101} pole figure (2*θ* = 25.224°) for TiO_2_ deposited on top of LAO substrate. In the color scale bar red (blue) indicates high (low) counts. (b) Azimuthal scans for LAO {101} at *χ* ∼ 45° and for anatase {101} at *χ* ∼ 68.2° and *χ* ∼ 41.0°.

The in-plane texture of the TiO_2_ film on LAO substrate was investigated performing an anatase {101} pole figure by setting 2*θ* = 25.224°. In Fig. 2a it is possible to observe four sharp peaks centered at *χ* = 68.2° that is exactly the interplanar angle between {001} and {101} anatase planes. These four poles make 90° with respect to each other and reveal a single in-plane orientation of the anatase {001} crystals. The weaker shadowed peaks that occur about 41° in Fig. 2a suggest that anatase crystals present tilted domains. This result could not be compared with any report in the literature since the authors did not find previous works showing pole figures of TiO_2_ films grown on LAO (100). For example, the work of Lotnyk *et al.* discussed above, only presented pole figures of TiO_2_ films on LAO (110) and on yttria-stabilized zirconia. Following, these tilted anatase domains are investigated in more detail using specific scans.

Azimuthal scans for *χ* = 68.2° at 2*θ* = 25.224° and *χ* = 45.0° at 2*θ* = 33.397° are shown in Fig. 2b. The first and second scans select anatase {101} and LAO {101} planes, respectively. As can be observed, there is no offset between {101} peaks of the anatase and LAO, implying that the anatase lattice is not rotated about the [001] substrate normal vector, which is in agreement with an earlier report.^36^ The ordering of this plane is due to the excellent matching between the base of the anatase conventional unit cell tetragon, and face of the cubic LAO unit cell. The difference of lattice parameters between then is only 0.2%.^26^ The FWHM of {101} peaks is 0.6° for LAO and 1.1° for anatase. This result implies in a very small difference of the in-plane orientation between anatase crystallites, *i.e.* a very low mosaicity of the anatase crystals in the film.

In the pole figure of Fig. 2a there are also weak spots at *χ* = 41.0°, which are explained by the growth of out-of-plane oriented anatase {112} grains already observed by the small peak in Fig. 1a. These spots are rotated by 45° in respect to the anatase {101} ones at *χ* = 68.2°. The azimuthal curve at *χ* = 41.0° in Fig. 2b shows that the anatase {112} grains are actually split by 3° in two peaks with a rotation of 43.5° and 46.5° over LAO [001] axis. The presence of anatase grains with such in-plane orientation on TiO_2_ epitaxial growth over LAO have not been previously reported. Therefore, we were encouraged to propose a possible atomic arrangement of anatase {112} planes in the TiO_2_ film grown on LAO.

**Fig. 3:**
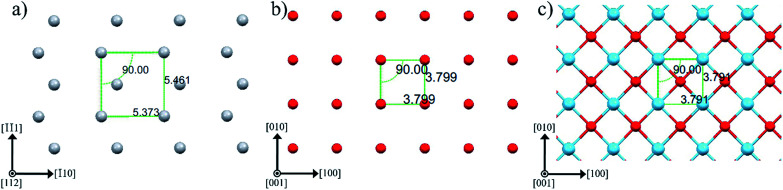
Atomic arrangement of (a) anatase (112), (b) anatase (001) and (c) LAO (001) planes. The atoms Ti, O and La are depicted by gray, red and blue spheres, respectively. These images were created making use of Mercury software.^56–58^

Fig. 3 sketches anatase (112), anatase (001) and LAO (001) planes. In this figure the atomic similarity between anatase (001) and LAO (001), that gives rise to the low lattice mismatch of 0.2%, becomes clear. If anatase (112) is rotated by 45° the square formed by Ti atoms, indicated in the figure, will fit to the anatase (001) plane or likewise to the LAO (001) plane. In anatase [1̄10] direction, the lattice mismatch would be close to zero and, in the direction [1̄1̄1], it would be approximately 1.6%. Such low values support the 45° rotation of anatase (112) and reasonably agrees with the results in Fig. 2b, where rotations of 43.5° and 46.5° were found. The divergence from 45° is probably related to lattice relaxation processes.

**Fig. 4:**
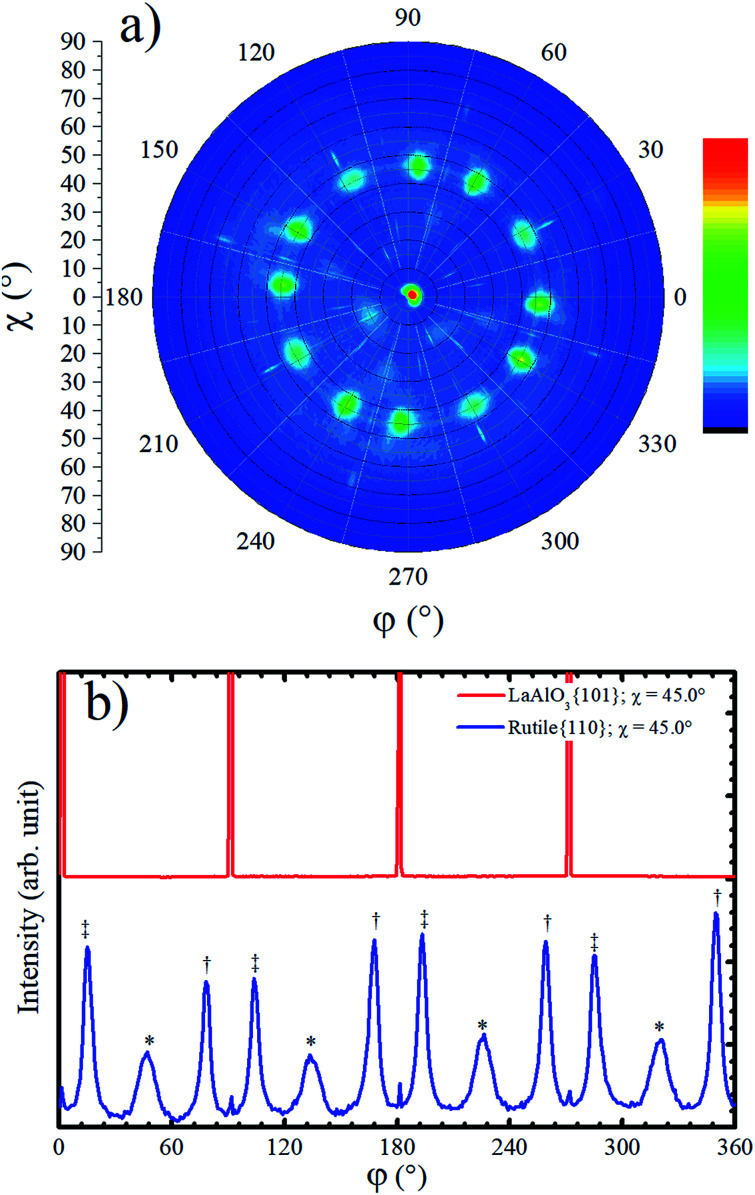
(a) Rutile {110} pole figure carried out at 2*θ* = 27.434° for a TiO_2_/LAO film. In the color scale bar red (blue) indicates high (low) counts. (b) Azimuthal scans at *χ* ∼ 45.0° for LAO {101} and for rutile {110}. The three different symbols in (b) indicate peaks related to three different arrangements of rutile crystallites.

Although the XRD Bragg–Brentano measurement in Fig. 1a has not shown any evidence for the presence of rutile phase in the TiO_2_/LAO film, a rutile {110} pole figure was measured, and twelve peaks centered at *χ* = 45.0° were observed, as shown in Fig. 4a. Such diffraction peaks are attributed to rutile crystallites with 〈100〉 out-of-plane growth direction, since rutile {100} planes make 45° with {110} ones. The absence of {*h*00} peaks in Bragg–Brentano measurements is probably due to the low amount of rutile phase in the TiO_2_ film combined with extinction effects. The observation of rutile in the pole figure only became possible due to the much higher structural factor of {110} planes in respect to any {*h*00} plane. For instance, the rutile (200) plane has the highest structural factor among the rutile {*h*00} planes and it is roughly two and a half times smaller^40^ than the rutile (110) structural factor.

The fraction of anatase (*f*_A_) in the TiO_2_/LAO sample is estimated using the following equation,^41^1
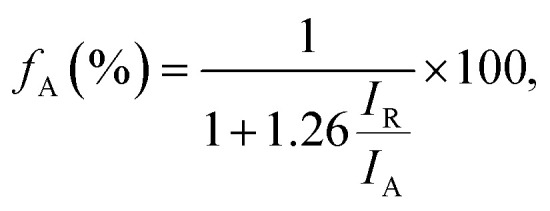
where, *I*_R_ and *I*_A_ are the intensities for the strongest anatase and rutile reflections, which are (101) and (110) reflections, respectively. The *I*_R_ and *I*_A_ values are obtained from the brightest spot in Fig. 4a and 2a, respectively. We verified from eqn (1) that the grown film is predominantly composed of anatase, with up to 4% rutile. It is important to highlight here, that in previous reports, in which the growth of single phase anatase films on LAO was claimed, the absence of rutile crystallites was not confirmed by inspection of rutile {110} pole figures.^26,36,37,42,43^ The present results demonstrate that Bragg–Brentano XRD measurement only may not be sufficient to rule out the presence of small concentrations of rutile crystallites in allegedly single-phase anatase films.

Rutile {110} azimuthal scan for *χ* = 45.0° is shown in Fig. 4b. The positions of the peaks can be explained on basis of the geometry of the crystal cut interface between LAO and rutile: the first intense peak, relatively narrow, marked with a double cross symbol, corresponds to the diffractions from the rutile (110) planes associated to crystallites that match the diagonal of their rectangular basis *a* × *c* (5.46 Å) with cube diagonal *a* × *a* (5.36 Å) of LAO lattice. In the unstrained crystal condition, this should correspond to a 12.21° rotation, which is roughly the angle between LAO {101} peaks (see Fig. 4b) and rutile {110} peaks marked with double cross symbol. The clockwise and counterclockwise rotations provide the single and double cross marked peaks. The peaks labelled with an asterisk, correspond to the {110} rutile planes of crystallites grown with a rotation of 45° of the in-plane *a* axis, [100] direction, relative to the *a* axis of LAO. The poorer matching of these ones explains the lower intensity and the broader FWHM of these peaks. Then, the fourfold rotation symmetry of the [001] axis of LAO can explain the twelve {110} rutile peaks observed in the pole figure and azimuthal scan of Fig. 4. The small peaks at 0°, 90° and repeated each 90° are not rotated in respect to the substrate and probably correspond to diffraction signal related to the anatase 〈001〉 grains.

Due to the high similarity between atomic arrangements of LAO (001) and anatase (001) planes, the in-plane orientation of rutile grains discussed above is also valid if considered the growth of rutile (100) on top of anatase (001).

**Fig. 5:**
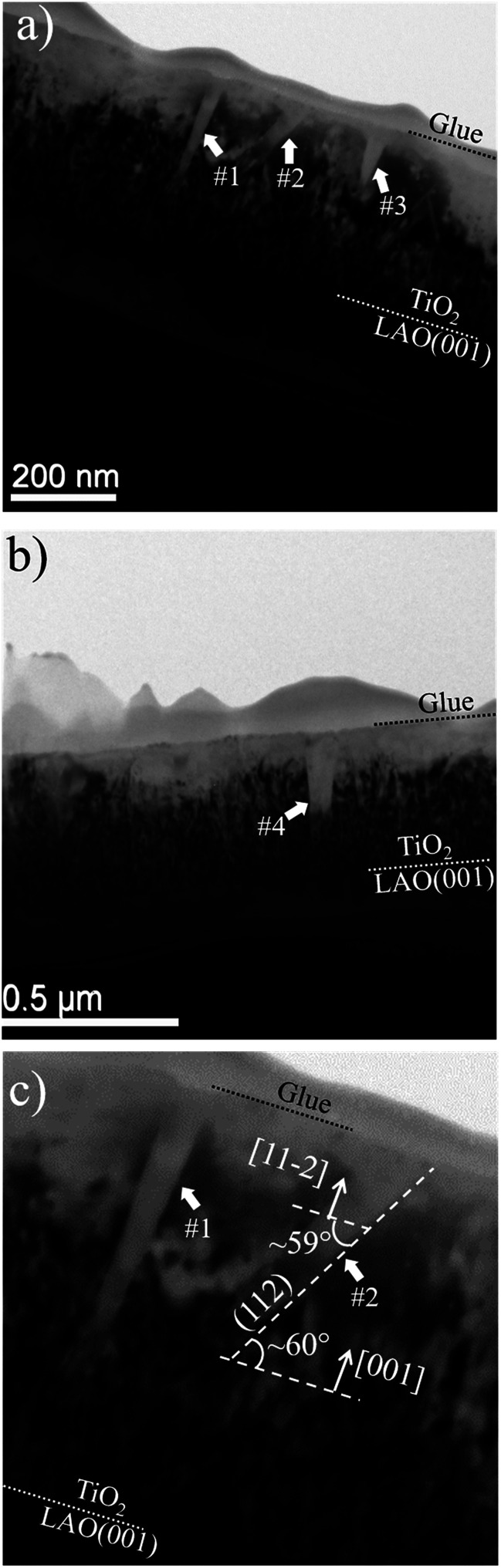
Bright field TEM images of different regions of the sample are shown in (a) and (b). The dotted lines indicate the interfaces between substrate, TiO_2_ film and the glue used during sample preparation for TEM. (c) Displays a zoom of regions marked as #1 and #2 in (a).

In order to investigate the local structural and morphological aspects of the films, TEM analysis were performed. Fig. 5 presents TEM images of different regions of the TiO_2_/LAO sample in which two types of columnar structures are observed that are possibly related to twins; (i) almost perpendicular or (ii) tilted with respect to the substrate surface. Similar structures associated to twins were also observed in silver thin films.^44^ To the best of our knowledge, twin structures were not previously observed in TiO_2_ films grown on LAO. In the work of H. Xu *et al.*,^36^ XRD of TiO_2_/LAO films did not show the anatase (112) peak and in agreement no twin structure was observed in the TEM images. The structures in Fig. 5 are indicated and numbered as #1, #3 and #4, corresponding to type (i) and #2, corresponding to type (ii). The two first (#1 and #2) are better presented in Fig. 5c, where it is possible to note a ∼60° tilt of #2 in respect to the substrate surface. This angle is very close to the angles between anatase (112) and (001) planes, and between anatase (112) and (112̄) planes, where the calculated values are 60.53° and 58.93°,^45^ respectively. Therefore, we can consider the twin plane being anatase (112), the columnar structure growing in anatase [112̄] direction and the TiO_2_ portion surrounding this columnar grain growing in anatase [001] direction, as indicated in Fig. 5c. This consideration is supported by the fact that the major part of the sample presents [001] orientation (see XRD results in Fig. 1a). The other structures (#1, #3 and #4) do not show the ∼60° inclination because the respective columnar grains are ∼90° in-plane rotated in respect to #2. In accordance with azimuthal curves in Fig. 2b that showed four-fold symmetry for anatase (112) grains.

The formation of anatase {112} twin interfaces in TiO_2_/LAO sample is probably the source for the growth of the rutile crystallites, which presence was verified by the pole figure in Fig. 4a. It was previously observed in literature that rutile crystallites are likely to nucleate at {112} twin interfaces formed in nanocrystalline titania.^46,47^

**Fig. 6:**
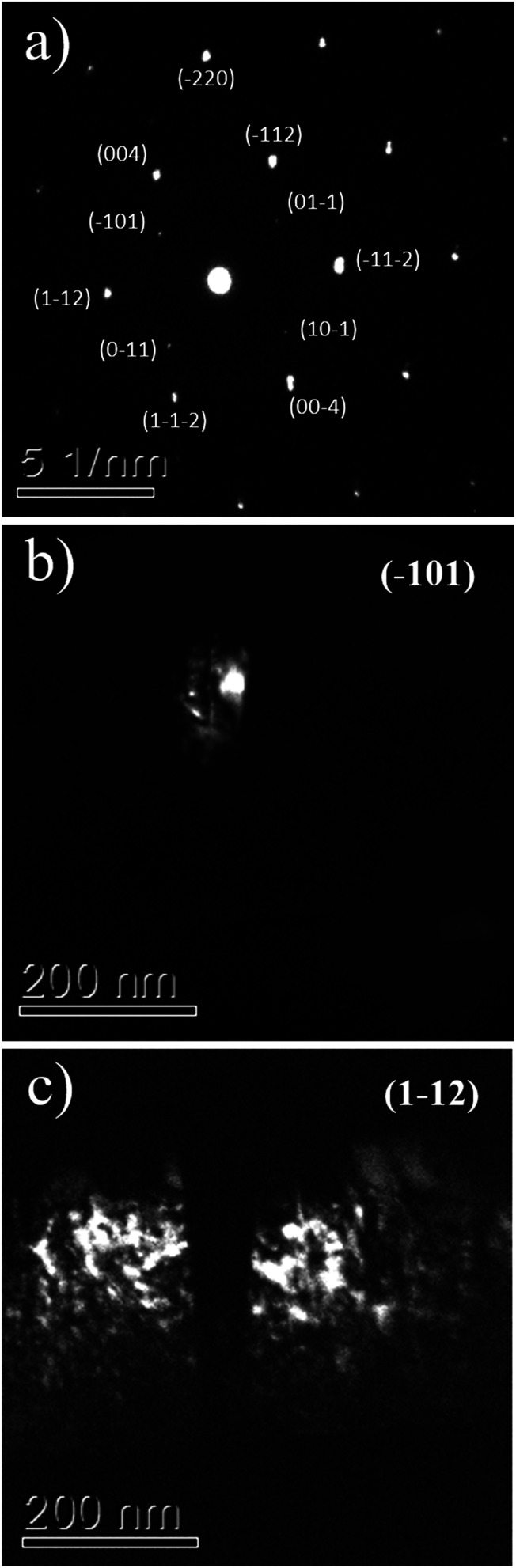
The TEM SAED pattern of the TiO_2_/LAO film is shown in (a), while (b) and (c) display the dark field images associated to diffractions (−101) and (1−12).

The interpretation that the columnar structures shown in Fig. 5 have a different crystalline orientation than the material surrounding it, is confirmed by dark field imaging of TiO_2_ grains encompassing a twin. In Fig. 6a it is displayed a SAED pattern took from the region shown in Fig. 5b. The diffraction spots are the expected ones for zone axes (ZA) near to anatase [110] and [111]. Dark field images for the diffracted beams (−101) and (1−12) are displayed in Fig. 6b and c, respectively. Wherein (−101) and (1−12) diffracted beams belong to anatase [110] and [111] ZA, respectively. The dark field images show that the columnar structure is close to the [110] ZA and the TiO_2_ grains out of this columnar structure are near to the [111] ZA. Therefore, the columnar structure is misoriented in respect to the rest portion of the TiO_2_ film.

Comparing these results with the literature, it is important to mention that Jeong and co-workers^20^ were the first to report the growth of single phase anatase onto LAO using reactive sputtering. They claim, that this technique has much better perspective for applications than MBE and PLD. In the growth they have used higher substrate temperatures (750 °C) and deposition power (250 W) than the presently reported. Their analysis is restricted to multiple axis X-ray diffraction and atomic force microscopy measurements. Very narrow peaks in the (004) anatase rocking curves (Δ*θ* = 0.09°) were obtained, revealing very good quality of the anatase crystals. No pole figure, electron microscopy nor optical results were presented. No mention was made to a search for rutile phase or for twin formation.

In a related paper of the same group,^30^ spectroscopic ellipsometry results of films prepared by reactive sputtering using the same deposition system and under similar conditions were analyzed. In the Optical results section we have compared in detail their results with ours.

Relevant results concerning the anatase growth onto LAO by reactive sputtering were also reported by Wang *et al.*^34^ They combined advanced transmission electron microscopy with high-precision first-principles calculation to relate the crystalline structure of TiO_2_/LAO interface to its electronic properties. They confirm that the rf magnetron can produce high quality anatase crystallites on the top of LAO. Similarly to results of the anatase/LAO (001) epitaxy found in most literature reports, and also in our results, the anatase crystals display the [001] axis perpendicular to the LAO (001) substrate surface, as one could expect by the close match of the surface atomic spacing provided by this geometry. This was already shown in our Fig. 3b and c, and is responsible for the similarity of Fig. 1 in the Wang *et al.* publication^34^ and on the present report.

In spite of these similarities, provided by the epitaxy relation between film and substrate, there are important differences in the procedures and results. Differently from the procedure used on this manuscript where a pure metallic Ti target was used, Wang *et al.* have used a ceramic TiO_2_ target. Their films were produced at significantly higher temperatures (800 °C), and have been annealed at 1000 °C in air. Even though the FWHM of the diffraction peak is wide in the as grown condition, the annealing produced a significant decrease of the XRD peak width, indicating improvement of the anatase crystal quality with annealing.

XRD pole figures, are not present in this ref. 34. So it is not possible to directly verify if the coherence of the growth occurs in the whole surface or is restricted to the analysis points/axis and if the tilt of the anatase crystallites also occur on their films or not. Besides, the thicknesses of the film grown by them are significantly lower (∼50 nm) than the ones presented here (∼500 nm).

Due to texture effects the presence of small amounts of the rutile phase is difficult to detect in the conventional XRD analysis. The presence of rutile on films grown on LAO could not be detected from our TEM or from the regular XRD analysis. Also the optical results of the films on LAO present no clear indication that this phase is present. This proved the analysis using pole figures to be important.

### SiO_2_/Si(100) substrate

**Fig. 7:**
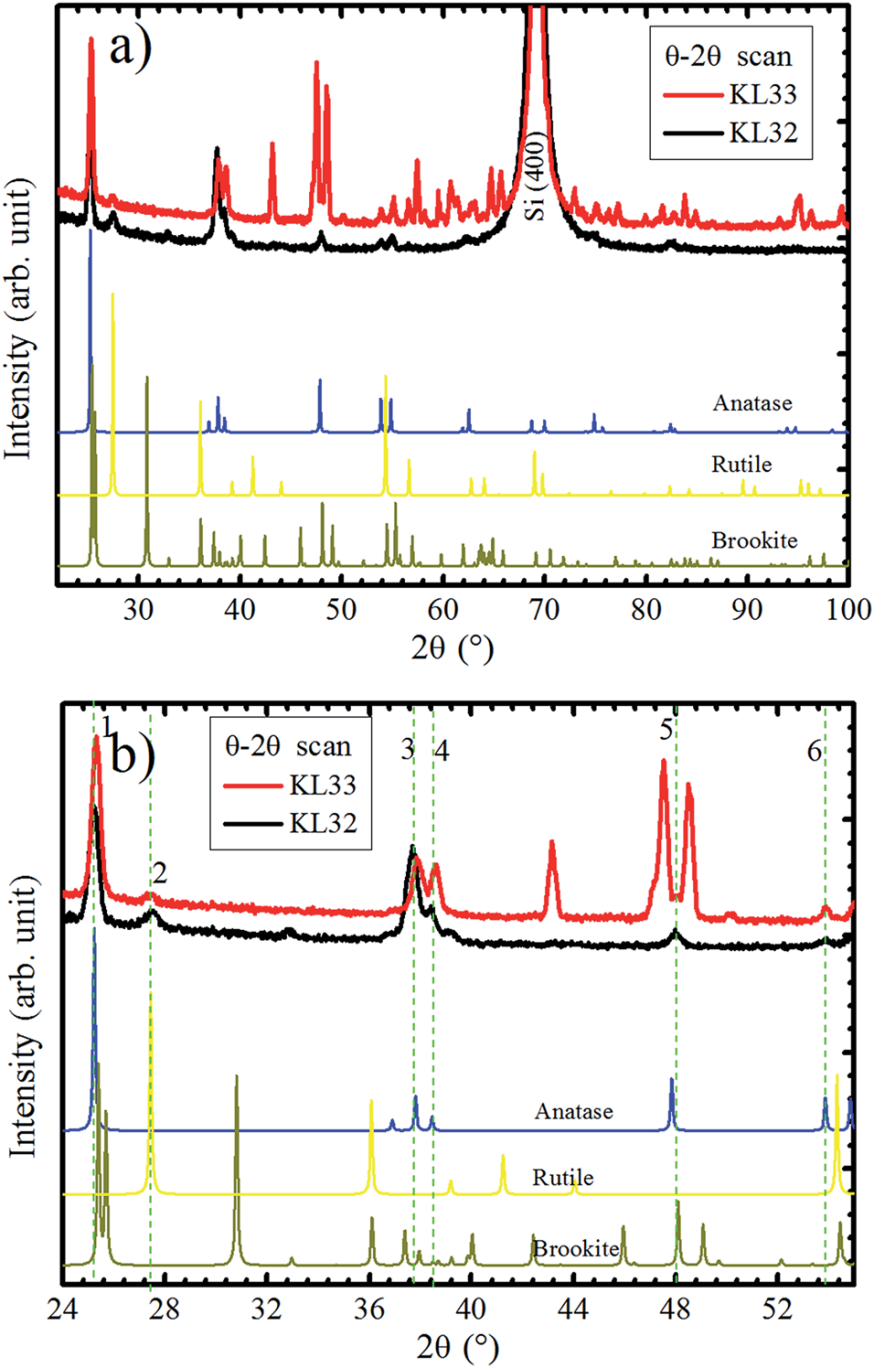
(a) XRD experiments in Bragg–Brentano mode of TiO_2_/SiO_2_/Si(100) samples. Samples KL32 (black curve) and KL33 (red curve), correspond to interrupted and continuous deposition O_2_ flows, respectively. (b) Zoom in of 2*θ* axis from 24 to 55° with the six XRD coincident peaks for both samples indicated by vertical dashed lines. In the bottom of the figures, reference diffractograms of anatase,^45^ brookite,^45^ and rutile^40^ are given for comparison.

In order to verify the substrate influence on the growth of TiO_2_ films, this oxide was deposited on SiO_2_/Si(100). Additionally, we investigated the influence of the O_2_ atmosphere variation on the structure of TiO_2_ films grown on SiO_2_. Fig. 7a shows XRD patterns, obtained in Bragg–Brentano mode, for TiO_2_ films deposited with continuous (KL33) and discontinuous (KL32) O_2_ supply. These results are identical to the ones obtained for TiO_2_ grown on a-SiO_2_ (results not showed here). In agreement with previous characterization by Raman spectroscopy and XRD, the TiO_2_ films are formed by the anatase, rutile and brookite phases, with the last two phases in a minor concentration.^24^ There is a clear reduction in the number of peaks from sample KL33 to KL32, therefore, the sample KL32 displays a less misoriented growth than the KL33 one. This is an evidence of structural modifications caused by the modulation of O_2_ supply during deposition. Moreover, it demonstrates that the TiO_2_ growth on SiO_2_ is kinetically controlled by O_2_ concentration. On the other hand, the non-dependence on the O_2_ supply and the high texture presented by TiO_2_ films grown on LAO are evidences that such growth is less affected by the kinetics of the process and that it is actually thermodynamically governed by the free energy of formation of TiO_2_ layers on LAO. The kinetically/thermodynamically controlled growth of nanomaterials has been discussed regarding different deposition systems, *e.g.* chemical synthesis of Rh–Pd alloy nanocrystals^48^ and electrodeposition of Cu_2_O films.^49^

In Fig. 7a, even sample KL32 does not show such a strong out-of-plane preferential orientation as the TiO_2_ layers grown on LAO, which highlights the role played by the substrate on determining the TiO_2_ film structure.

A zoom in 2*θ* axis from 24° to 55° could bring some additional information, as shown in Fig. 7b. The XRD peaks that are observed in both samples are numbered from 1 to 6. The 2*θ* positions of peaks 1, 3 and 4 for KL33 are displaced from the ones observed for KL32, indicating different lattice parameters for grains grown under continuous and discontinuous O_2_ supply. Therefore, at least in one of the deposition conditions, part of the TiO_2_ grains are under lattice strain. On the other hand, peaks 2, 5 and 6 show same positions for both samples, which means equal interplanar spacing. These diffractions are assigned to rutile (110), brookite (321) and anatase (105) planes, respectively. These peaks with equal interplanar spacing for both samples probably emerge due to structural relaxation processes, *e.g.* crystal twins and phase transformation.^50,51^

**Fig. 8:**
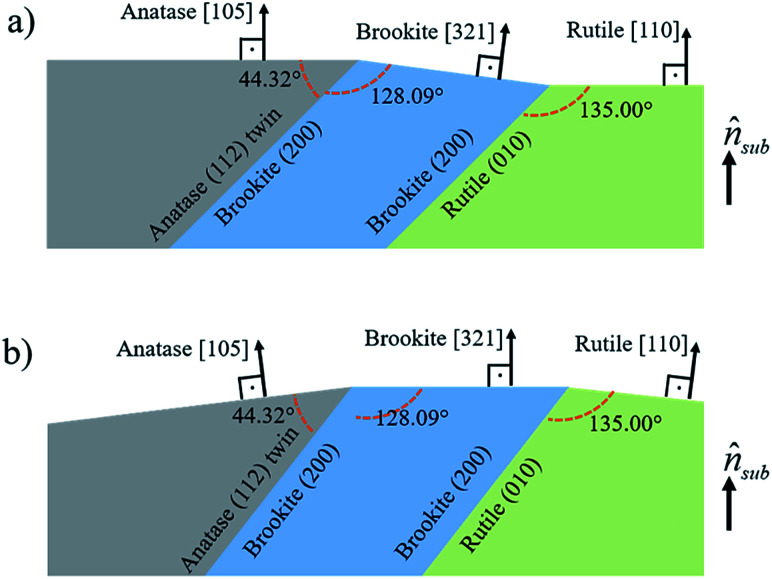
Sketch of anatase [105], brookite [321] and rutile [110] grains interrelated by an anatase (112) twin followed by formation of brookite (200) and rutile (010) planes. In (a) anatase [105] and rutile [110] and in (b) brookite [321] grow parallel to *n̂*_sub_.

In Fig. 8, it is sketched the growth of grains of the three TiO_2_ phases, anatase [105], brookite [321] and rutile [110], considering the phase transformation anatase–brookite–rutile already discussed in the literature,^46,47,52^ which occurs *via* anatase {112} twin formation followed by brookite (200) and rutile (010) growth. Assuming that the planes anatase (112) twin, brookite (200) and rutile (010) are parallel to each other will imply that the brookite [321] vector in Fig. 8a and anatase [105] and rutile [110] vectors in Fig. 8b not being parallel to the substrate normal (*n̂*_sub_). Intermediary situations in which none of the three directions are parallel to *n̂*_sub_ are also possible. This interpretation is in agreement with XRD rocking curves of brookite (321), rutile (110), and anatase (105) peaks that presented a broad FWHM of ∼14° (results not shown) indicating that those crystals are not all growing parallel to *n̂*_sub_. Therefore, the growth scheme in Fig. 8 supports the hypothesis of a structural relaxation mediated by crystal twining that gives rise to brookite and rutile formation in both conditions of O_2_ flux. The observation and modeling of the anatase {112} twin formation can be an important step towards understanding the mechanisms responsible for the growth of brookite and rutile phases in predominantly anatase thin films.

### Optical results

**Fig. 9:**
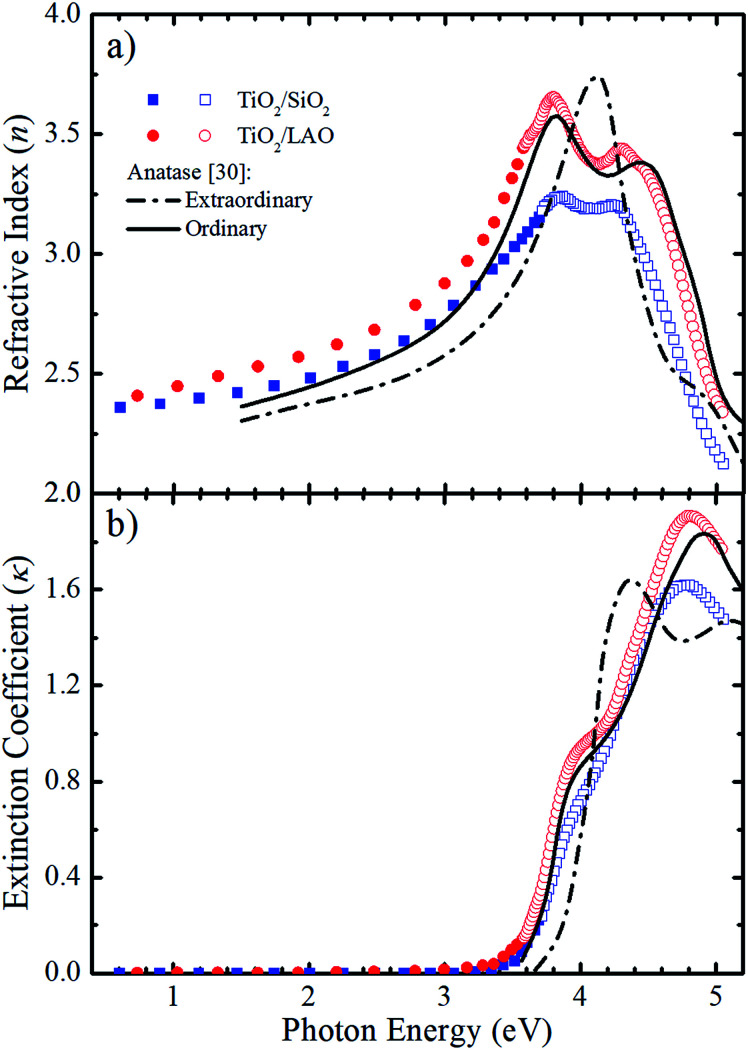
Comparison between the calculated (a) *n* and (b) *κ* values for both TiO_2_ on a-SiO_2_ (blue) and LAO (red) of samples grown under continuous O_2_ flow with the values reported by Jellison Jr *et al.*^30^ (black lines) for anatase bulk crystals. The calculated values were obtained using transmittance (full symbols) and ellipsometry (open symbols) measurements.

With emphasis on the study of the influence of SiO_2_ and LAO substrates on the growth of TiO_2_ films using the RF sputtering technique, we analyzed the optical properties of the produced layers by means of optical transmittance and spectroscopic ellipsometry measurements. Fig. 9 displays the refractive index (*n*) and the extinction coefficient (*κ*) determined for TiO_2_ deposited on LAO and a-SiO_2_. The full symbols, in the weakly absorbing region, represent values obtained *via* transmittance measurements followed by calculations based on the Cisneros' method.^53^ The values depicted by open symbols were determined from ellipsometric measurements and using Tauc–Lorentz analytical functions.^54^

The refractive index and extinction coefficients of bulk anatase crystals obtained by Jellison *et al.*^30^ are also plotted in Fig. 9 for comparison. The continuous lines represent the values for light propagation along the anatase *c*-axis (ordinary), while the dot-dash lines for light propagation perpendicular to the *c*-axis (extraordinary).

Comparing the curves in Fig. 9a, it can be noticed that the *n* values for the TiO_2_/LAO sample behave very similarly to the expected for the ordinary direction of the TiO_2_ anatase phase. At high photon energies, the two peaks around 3.8 and 4.4 eV, related to the ordinary axis of the anatase phase TiO_2_ crystal, are reproduced quite well by the TiO_2_/LAO sample. On the other hand, the TiO_2_/SiO_2_*n* curve at photon energies higher than 3.5 eV does not fit so well with the *n* curve for ordinary propagation of light measured in bulk anatase by Jellison Jr *et al.*^30^ The results of Fig. 9a, show an agreement between the observations of XRD of Fig. 1, 2 and 7, and the refractive index, which shows a behavior very close to anatase. Nevertheless, the optical data did not reveal the presence of the small amount of the rutile and brookite phases in films deposited in both substrates. In Fig. 9b the shoulder at about 3.9 eV and the maximum around 4.9 eV in the anatase *κ*_ord_ curve are observed in both samples. However, the energy position and intensity of the maximum is better reproduced by the TiO_2_/LAO sample, which reinforces that TiO_2_ grown on LAO is highly crystalline, the anatase *c*-axis being perpendicular to the substrate surface.

Therefore, it is clear that the refractive index and extinction coefficient of the samples grown on both substrates are compatible with the strong predominance of the anatase phase. However, due to structural modifications caused by the use of distinct substrates, the electronic properties are different for TiO_2_ layers grown on LAO and a-SiO_2_. Analyzing the results of previous investigations and comparing the optical properties of crystalline and polycrystalline anatase by Tanemura and co-workers,^55^ it becomes evident that the present optical results reinforce the high crystallinity of the TiO_2_/LAO sample, observed by XRD in Fig. 1 and 2. For the TiO_2_/SiO_2_, the optical characteristics are also similar to the ones of anatase crystals, but are also compatible with the more diffuse aspect of the X-ray pole figures^24^ and high misorientation (Fig. 7), related to a more random orientation of the crystallites.

**Fig. 10:**
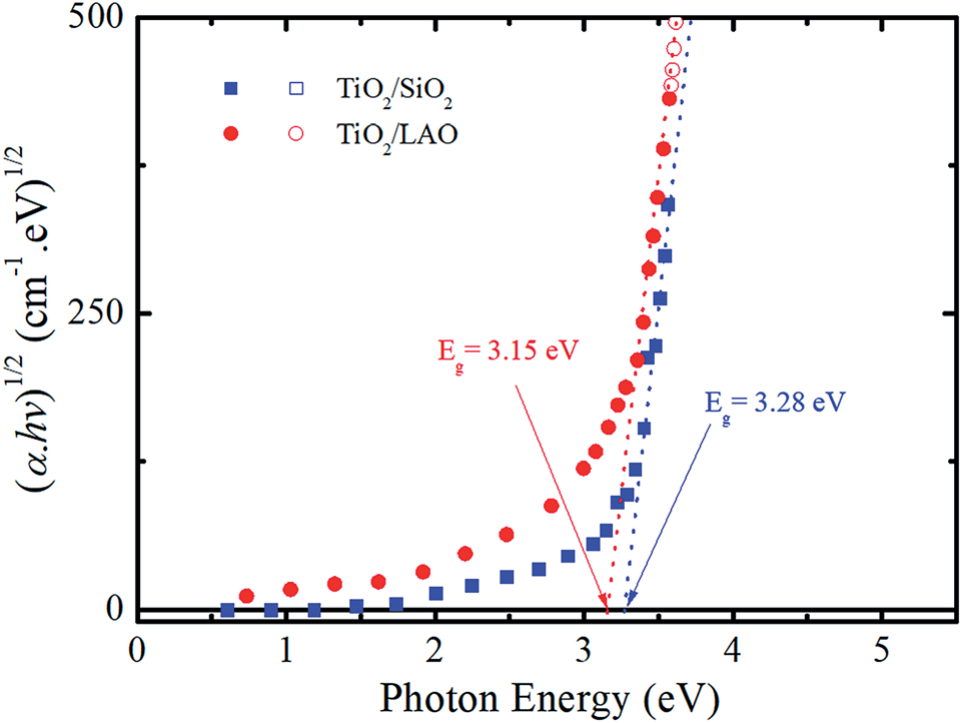
Tauc plot of (*αhν*)^1/2^*versus* photon energy (*hν*) for both TiO_2_/LAO and TiO_2_/SiO_2_ samples.

From the extinction coefficient data of Fig. 9b, the absorption coefficient (*α*) was calculated for each sample and then fitted by the Tauc's relation,^54^ (*αhv*)^1/2^ = *A*(*hv* − *E*_g_), that holds for indirect bandgap materials. In this equation *hv* is the photon energy, *A* is a constant and *E*_g_ is the band gap. The determined *E*_g_ values from Fig. 10 are 3.15 and 3.28 eV for TiO_2_/LAO and TiO_2_/SiO_2_ samples, respectively. It is shown that the TiO_2_ layers on LAO presents a slightly higher absorption in the visible than TiO_2_ layers deposited onto SiO_2_.

## Conclusions

In summary, the structural properties of TiO_2_ films grown by reactive sputtering on LAO, SiO_2_/Si(100) and SiO_2_ substrates using continuous and modulated oxygen flux were investigated using a combination of techniques. On the analysis presented here, XRD pole figures, and the optical combined T & R and ellipsometry probed large areas, and large volumes, of the films making the conclusions more robust when concerning large area applications. In contrast, the details in the structure were probed using transmission electron spectroscopy. The films are predominantly composed by the anatase phase, especially the ones deposited onto LAO, in which the percentage of anatase was estimated as 96%. On both crystalline and amorphous substrates, the growth of rutile and brookite phases were shown to be mediated by formation of anatase {112} twin interfaces. The TiO_2_ layers grown on LAO presented high crystallinity with in-plane and out-of-plane orientations, for both the anatase crystals and also for crystallites of the minor rutile phase. The pole figures and azimuthal scans were especially useful in the present analysis, and allowed a detailed investigation of the structure of TiO_2_ films onto LAO substrate. These techniques have clearly shown the epitaxial growth of the predominant anatase phase and the tilt in orientation produced by twin defect formation. Also, these techniques allowed the detection and an unpreceded detailed texture analysis of the tiny rutile phase. Meanwhile, TiO_2_ deposited in SiO_2_ substrates are polycrystalline with some orientation texture. Such structural differences are responsible for distinct optical electronic properties of TiO_2_ layers grown on LAO and SiO_2_, as observed from optical transmittance and ellipsometric measurements.

## Conflicts of interest

There are no conflicts to declare.

## Supplementary Material

## References

[cit1] Fujishima A., Honda K. (1972). Nature.

[cit2] Linsebigler A. L., Linsebigler A. L., Yates Jr J. T., Lu G., Lu G., Yates J. T. (1995). Chem. Rev..

[cit3] Henderson M. a. (2011). Surf. Sci. Rep..

[cit4] Hashimoto K., Irie H., Fujishima A. (2005). Jpn. J. Appl. Phys..

[cit5] Li L.-H., Kong Y.-M., Kim H.-W., Kim Y.-W., Kim H.-E., Heo S.-J., Koak J.-Y. (2004). Biomaterials.

[cit6] Paz Y., Luo Z., Rabenberg L., Heller a. (1995). J. Mater. Res..

[cit7] Feng X., Shankar K., Varghese O. K., Paulose M., Latempa T. J., Grimes C. a. (2008). Nano Lett..

[cit8] Wang D., Choi D., Li J., Yang Z., Nie Z., Kou R., Hu D., Wang C., Saraf L. V., Zhang J., Aksay I. a., Liu J. (2009). ACS Nano.

[cit9] Bera M. K., Mahata C., Chakraborty a. K., Nandi S. K., Tiwari J. N., Hung J.-Y., Maiti C. K. (2007). Semicond. Sci. Technol..

[cit10] Liu L., Zhao H., Andino J. M., Li Y. (2012). ACS Catal..

[cit11] O'Regan B., Grätzel M. (1991). Nature.

[cit12] Hadjiivanov K. I., Klissurski D. G. (1996). Chem. Soc. Rev..

[cit13] Scanlon D. O., Dunnill C. W., Buckeridge J., Shevlin S. A., Logsdail A. J., Woodley S. M., Catlow C. R. A., Powell M. J., Palgrave R. G., Parkin I. P., Watson G. W., Keal T. W., Sherwood P., Walsh A., Sokol A. A. (2013). Nat. Mater..

[cit14] Arrouvel C., Digne M., Breysse M., Toulhoat H., Raybaud P. (2004). J. Catal..

[cit15] Chen J. S., Tan Y. L., Li C. M., Cheah Y. L., Luan D., Madhavi S., Boey F. Y. C., Archer L. a., Lou X. W. (2010). J. Am. Chem. Soc..

[cit16] Hengerer R., Kavan L., Krtil P., Gratzel M. (2000). J. Electrochem. Soc..

[cit17] Luttrell T., Halpegamage S., Tao J., Kramer A., Sutter E., Batzill M. (2014). Sci. Rep..

[cit18] Olson C. L., Nelson J., Islam M. S. (2006). J. Phys. Chem. B.

[cit19] Bin Wu H., Chen J. S., Hng H. H., Wen X., Lou D. (2012). Nanoscale.

[cit20] Jeong B. S., Budai J. D., Norton D. P. (2002). Thin Solid Films.

[cit21] Jeong B.-S., Norton D. P., Budai J. D. (2003). Solid-State Electron..

[cit22] Jeong B., Norton D. P., Budai J. D., Jellison G. E. (2004). Thin Solid Films.

[cit23] Lee G.-H., Kim M.-S. (2010). Electron. Mater. Lett..

[cit24] Pereira A. L. J., Lisboa Filho P. N., Acuña J., Brandt I. S., Pasa A. a., Zanatta A. R., Vilcarromero J., Beltrán A., Dias da Silva J. H. (2012). J. Appl. Phys..

[cit25] Pereira A. L. J., Gracia L., Beltrán A., Lisboa-Filho P. N., Silva J. H. D., Andrés J. (2012). J. Phys. Chem. C.

[cit26] Murakami M., Matsumoto Y., Nakajima K., Makino T., Segawa Y., Chikyow T., Ahmet P., Kawasaki M., Koinuma H. (2001). Appl. Phys. Lett..

[cit27] Schmidt D. A., Ohta T., Yu Q., Olmstead M. A. (2006). J. Appl. Phys..

[cit28] Park B. H., Huang J. Y., Li L. S., Jia Q. X. (2002). Appl. Phys. Lett..

[cit29] Kim D., Kim W.-S., Kim S., Hong S.-H. (2014). ACS Appl. Mater. Interfaces.

[cit30] Jellison G. E., Boatner L. A., Budai J. D., Jeong B.-S., Norton D. P. (2003). J. Appl. Phys..

[cit31] Lotnyk A., Senz S., Hesse D. (2007). Thin Solid Films.

[cit32] Silva V. F., Bouquet V., Deputier S., Lebullenger R., Guilloux-Viry M., Silva V. L., Santos I. M. G., Perrin A., Weber I. T. (2017). J. Nanosci. Nanotechnol..

[cit33] Weng X., Fisher P., Skowronski M., Salvador P. A., Maksimov O. (2008). J. Cryst. Growth.

[cit34] Wang Z., Zeng W., Gu L., Saito M., Tsukimoto S., Ikuhara Y. (2010). J. Appl. Phys..

[cit35] Baía I., Quintela M., Mendes L., Nunes P., Martins R. (1999). Thin.

[cit36] Xu H., Feng X., Luan C., Ma J. (2016). Scr. Mater..

[cit37] Krupski K., Sanchez A. M., Krupski A., McConville C. F. (2016). Appl. Surf. Sci..

[cit38] Kennedy R. J., Stampe P. A. (2003). J. Cryst. Growth.

[cit39] Pulsed Laser Deposition of Thin Films, ed. D. B. Chrisey and G. K. Hubler, Wiley, New York, 1994

[cit40] Baur W. H. (1956). Acta Crystallogr..

[cit41] Spurr R. A., Myers H. (1957). Anal. Chem..

[cit42] Ciancio R., Vittadini A., Selloni A., Arpaia R., Aruta C., Granozio F.
M., di Uccio U. S., Rossi G., Carlino E. (2013). J. Nanopart. Res..

[cit43] Breeson A. C., Sankar G., Goh G. K. L., Palgrave R. G. (2016). Phys. Chem. Chem. Phys..

[cit44] Tomov I., Adamik M., Barna P. B. (2000). Thin Solid Films.

[cit45] Djerdj I., Tonejc A. M. (2006). J. Alloys Compd..

[cit46] Penn R. L., Banfield J. F. (1999). Am. Mineral..

[cit47] Zhou Y., Fichthorn K. a. (2012). J. Phys. Chem. C.

[cit48] Yan Y., Zhan F., Du J., Jiang Y., Jin C., Fu M., Zhang H., Yang D. (2015). Nanoscale.

[cit49] Pelegrini S., Brandt I. S., Plá Cid C. C., Isoppo E. A., Viegas A. D. C., Pasa A. A. (2015). ECS J. Solid State Sci. Technol..

[cit50] Clevenger L. A., Mutscheller A., Harper J. M. E., Cabral C., Barmak K. (1992). J. Appl. Phys..

[cit51] Clausen B., Tomé C. N., Brown D. W., Agnew S. R. (2008). Acta Mater..

[cit52] Penn R. L., Banfield J. F. (1998). Am. Mineral..

[cit53] Cisneros J. I. (1998). Appl. Opt..

[cit54] Tauc J., Grigorovici R., Vancu A. (1966). Phys. Status Solidi.

[cit55] Tanemura S., Miao L., Jin P., K K., Terai A., Nabatova-Gabain N. (2003). Appl. Surf. Sci..

[cit56] Bruno I. J., Cole J. C., Edgington P. R., Macrae C. F., Pearson J., Taylor R. (2002). Acta Crystallogr., Sect. B: Struct. Sci..

[cit57] Macrae C. F., Edgington P. R., McCabe P., Pidcock E., Shields G. P., Taylor R., Towler M., van de Streek J. (2006). J. Appl. Crystallogr..

[cit58] Macrae C. F., Bruno I. J., Chisholm J. A., Edgington P. R., McCabe P., Pidcock E., Rodriguez-Monge L., Taylor R., van de Streek J., Wood P. A. (2008). J. Appl. Crystallogr..

